# Supplementary material for: Functional specialization of mPFC-BLA and mPFC-NAc pathways in affective state representation
Source: eLife. 2026 Aug 7;14:RP105528. doi: 10.7554/eLife.105528 (PMC13451018; doi:10.7554/eLife.105528)
Supplement: Peer review history

# Editor report: eLife Assessment

**DOI:** 10.7554/eLife.105528.5.sa0

**Authors:** Michelle Antoine

**Affiliations:** National Institute of Alcohol Abuse and Alcoholism United States

**Keywords:** Solid, Valuable

Du et al. present a **valuable** study examining neural activation in the medial prefrontal cortex (mPFC) subpopulations projecting to the basolateral amygdala (BLA) and nucleus accumbens (NAc) during behavioral tasks assessing anxiety, social preference, and social dominance. The strength of the evidence linking in vivo neural physiology to behavioral outcomes was considered **solid**. Overall, the reviewers felt that the revised work provides insight into how distinct mPFC→BLA and mPFC→NAc pathways influence anxiety, exploration, and social behaviors.

# Referee report: Reviewer #1 (Public review)

**DOI:** 10.7554/eLife.105528.5.sa1

[Editors' note: this version has been assessed by the Reviewing Editor without further input from the original reviewers. The authors have addressed the comments raised in the previous round of review.]

Summary:

It is well known that neurons in the medial prefrontal cortex (mPFC) are involved in higher cognitive functions such as executive planning, motivational processing and internal state mediated decision-making. These internal states often correlate with the emotional states of the brain. While several studies point to the role of mPFC in regulating behavior based on such emotional states, the diversity of information processing in its sub-populations remains a less explored territory. In this study, the authors try to address this gap by identifying and characterizing some of these sub-populations in mice using a combination of projection-specific imaging, function-based tagging of neurons, multiple behavioral assays and ex-vivo patch clamp recordings.

Strengths:

The authors targeted mPFC projections to the nucleus accumbens (NAc) and basolateral amygdala (BLA). Using the open field task (OFT), the authors identified four relevant behavioral states as well as neurons active while the animal was in the center region ("center-ON neurons"). By characterizing single unit activity and using dimensionality reduction, the authors show differentiated coding of behavioral events at both the projection and functional levels. They further substantiate this effect by showing higher sensitivity of mPFC-BLA center-ON neurons during time spent in the open arms of the elevated plus maze (EPM). The authors then pivoted to the three-chamber social interaction (SI) assay to show the different subsets of neurons encode preference of social stimulus over non-social. This reveals an interesting diversity in the function of these sub-populations on multiple levels. Lastly, the authors used the tube test as a manipulation of the anxiety state of mice and compared behavioral differences before/after in the OFT and social interaction tasks. This experiment revealed that "losers" of the tube test spend less time in the center of the open field while "winners" show a stronger preference for the familiar mouse over the object. Using patch-clamp experiments, the authors also found that "winners" exhibit stronger synaptic transmission in the mPFC-NAc projection while "losers" exhibit stronger synaptic transmission in the mPFC-BLA projection. Given the popularity of the tube test assay in rank determination, this provides useful insights into possible effects on anxiety levels and synaptic plasticity. Overall, the many experiments performed by the authors reveal interesting differences in mPFC neurons relative to their involvement in high or low anxiety behaviors, social preference and social rank.

Weaknesses:

The authors have addressed all comments.

# Referee report: Reviewer #2 (Public review)

**DOI:** 10.7554/eLife.105528.5.sa2

Summary:

The goal of this proposal was to understand how two separate projection neurons from the medial prefrontal cortex, those innervating the basolateral amygdala (BLA) and nucleus accumbens (NAc), contribute to the encoding of emotional behaviors. The authors record the activity of these different neuron classes across three different behavioral environments. They propose that, although both populations are involved in emotional behavior, the two populations have diverging activity patterns in certain contexts. A subset of projections to the NAc appear particularly important for social behavior. They then attempt to link these changes to the emotional state of the animal and changes in synaptic connectivity.

Strengths:

The behavioral data builds on previous studies of these projection neurons supporting distinct roles in behavior and extend upon previous work by looking at the heterogeneity within different projection neurons across contexts, this is important to understand the "neural code" within the PFC that contributes to such behaviours and how it is relayed to other brain structures.

Weaknesses:

The diversity of neurons mediating these projections and their targeting within the BLA and NAc is not explored. These are not homogeneous structures and so one possibility is that some of the diversity within their findings may relate to targeting of different sub-structures within BLA or NAc or the diversity of projection neuron subtypes that mediate these pathways. This is an important future direction for this work but does not detract from the main finding as reported.

# Author comment: Author response

**DOI:** 10.7554/eLife.105528.5.sa3

**Authors:** Chien-Hsien Lai, Gyeongah Park, Pan Xu, Xiaoqian Sun, Qian Ge, Zhen Jin, Sarah Betts, Xiaojie Liu, Qing-song Liu, Rahul Simha, Chen Zeng, Hui Lu, Jianyang Du

**Affiliations:** George Washington University Washington, DC United States; University of Tennessee Health Science Center Memphis United States; George Washington University Washington, DC Thailand; George Washington University Washington, DC United States; University of Tennessee Health Science Center Memphis United States; University of Tennessee Health Science Center Memphis United States; George Washington University Washington, DC United States; Medical College of Wisconsin Milwaukee United States; Medical College of Wisconsin Milwaukee United States; George Washington University Washington, DC United States; George Washington University Washington, DC United States; GEORGE WASHINGTON UNIVERSITY Washington United States; University of Tennessee Health Science Center Memphis United States

The following is the authors’ response to the previous reviews

**Public Reviews:**

**Reviewer #2 (Public review):**

Weakness:

The diversity of neurons mediating these projections and their targeting within the BLA and NAc is not explored. These are not homogeneous structures and so one possibility is that some of the diversity within their findings may relate to targeting of different sub-structures within BLA or NAc or the diversity of projection neuron subtypes that mediate these pathways. This is an important future direction for this work but does not detract from the main finding as reported. The electrophysiological data in Figure 7 have some experimental confounds that makes their interpretation challenging.

We thank the reviewer for these thoughtful comments. We fully agree that targeting different substructures within the BLA or NAc, as well as the diversity of projection neuron subtypes mediating these pathways, represents an important direction for future investigation. We will certainly explore these possibilities in future studies.

We have also removed the optogenetics and electrophysiology data, as they may introduce confounds. The removal of these data and figures does not affect our main conclusions.

**Recommendations for the authors:**

**Reviewer #2 (Recommendations for the authors):**

(a) The authors have improved the manuscript somewhat by refining their description of the results. However, the normalized EPSC experiments still do not make much sense. If you have a higher light intensity or LED duration the curve of the EPSC response will saturate earlier. Similarly, if you are in a highly, or poorly labeled slice or subregion of a slice then you will see responses emerge at different intensities based on the number of synapses labelled. There is no standardization in the way these experiments were performed, so performing some arbitrary post hoc normalisation does not correct for this. Similarly, they also place the fibreoptic manually above the slice each time. This makes it much harder to determine the actual light intensity delivered to the slice on a cell by cell and group by group basis.

I have reduced my public statement from significant experimental confounds, to some experimental confounds. But the way the experiments were performed does not allow the normalized data to really be interpretable. They still argue that normalized EPSCs are relatively larger. I don't even really understand what this means biologically.

The subsequent rise/decay and other measures is now better described. However, they note that the decay constant is larger. This means that the kinetics are slower, not enhanced, as they describe.

Again, we thank the reviewer for the careful advice. We recognize the limitations of the optogenetics and electrophysiology data and have therefore removed them to avoid potential confounds.
